# Significant Liver Fibrosis in Pre–Metabolic Dysfunction—Associated Alcohol-Relate Liver Disease Population: A Nationally Representative Analysis of Moderate Drinkers With Cardiometabolic Risk Factors

**DOI:** 10.1016/j.jceh.2026.103641

**Published:** 2026-08-18

**Authors:** Urmimala Chaudhuri, Makul Sharma, Erin Currey, Sangeeta Agrawal

**Affiliations:** ∗ Department of Internal Medicine, Wright State University Boonshoft School of Medicine, Dayton, OH, USA; † Department of Internal Medicine, Wright Patterson Medical Center, Dayton, OH, USA; ‡ Department of Gastroenterology, Wright State University, Dayton, OH, USA

**Keywords:** fibrosis screening, NHANES, MetALD, elastography

## Abstract

**Background/Aims:**

Metabolic dysfunction-associated alcohol–related liver disease (MetALD), a recently defined entity, represents the combination of metabolic dysfunction–associated steatotic liver disease (MASLD) and moderate to heavy alcohol consumption. While these thresholds mark the transition from MASLD to MetALD, the prevalence of significant liver fibrosis in individuals with metabolic dysfunction who consume alcohol below these levels, a “pre-MetALD” population, remains unknown. In this study, we estimated the burden of significant liver fibrosis within this pre-MetALD population using a nationally representative sample.

**Methods:**

We analyzed National Health and Nutrition Examination Survey 2021–2023 data. Adults aged ≥18 years with a body mass index ≥25 kg/m^2^ (≥23 for Asian participants), consuming 7–14 drinks per week with valid liver stiffness measurement (LSM) (interquartile range/median ≤0.30) and available Fibrosis-4 index (FIB-4) scores were included (N = 193). Cardiometabolic risk factor (CMRF) burden was scored using overweight/obesity, dysglycemia, and hypertension. Participants were categorized as having 1, 2, or 3 CMRFs. The primary outcome was an LSM ≥8 kPa; the secondary outcome was an LSM ≥12 kPa. Alcohol was categorized as low-moderate (7–10 drinks per week) or high-moderate (11–14 drinks per week). All analyses used survey weights.

**Results:**

The cohort had a mean age of 55.2 years (standard deviation: 15.4); 56.5% had hypertension and 30.1% had dysglycemia. Weighted prevalence of significant fibrosis was 15.8% (95% confidence interval [CI]: 9.2–22.4%), affecting approximately 1 in 6 participants. Fibrosis prevalence was similar across CMRF burden (15.0%, 16.4%, and 15.4% for 1, 2, and 3 CMRFs, respectively). A dose-response relationship was observed with alcohol intake, with significant fibrosis prevalence rising from 13.2% among those consuming 7–10 drinks per week to 18.0% among those consuming 11–14 drinks per week. Advanced fibrosis was present in 4.5% overall (95% CI: 1.9–7.1%). Notably, 40.9% of participants with significant fibrosis had a normal FIB-4 score (≤1.3). In the sensitivity analysis restricted to obese participants (n = 84), the prevalence rose to 23.2% (95% CI: 11.9–34.4%).

**Conclusion:**

Significant liver fibrosis affects approximately 1 in 6 adults with CMRFs who consume alcohol below the MetALD threshold. Nearly 41% of those with confirmed fibrosis had a normal FIB-4 score, underscoring the limitations of serum-based screening in this group. These findings support prospective evaluation of elastography-based screening strategies in the pre-MetALD population.

## Graphical abstract

**Figure:**
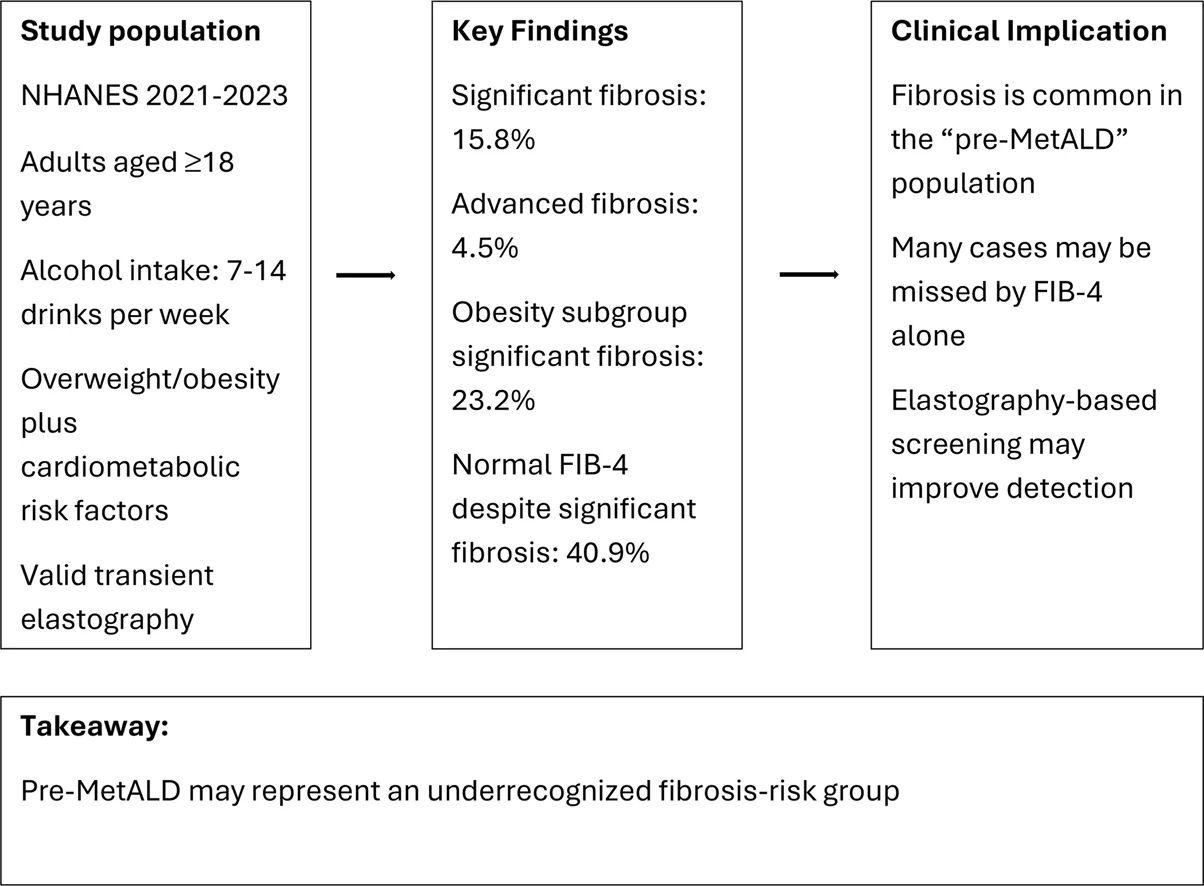

Metabolic dysfunction–associated alcohol-related liver disease (MetALD), a recently defined entity, represents the combination of metabolic dysfunction–associated steatotic liver disease (MASLD) and moderate to heavy alcohol consumption (140–350 g/week in women; 210–420 g/week in men). Following the multi-society Delphi consensus regarding nomenclature for steatotic liver disease (SLD), the terms MASLD, MetALD, and metabolic dysfunction–associated steatohepatitis were brought forth to address several of the challenges and limitations associated with the prior terminology.^1^ The introduction of MetALD as a new subclassification of SLD has helped raise awareness of disease prevalence and paved the way for therapeutic management and multidisciplinary approaches to patient care.^1^^,^^2^

MetALD arises from a complex interplay of intrahepatic and extrahepatic mechanisms, which include adipose tissue dysfunction, gut-liver axis dysbiosis, increased intestinal permeability, and disrupted bile acid signaling pathways.^3^ This in turn leads to hepatic lipotoxicity, cellular injury, immune activation, and subsequent fibrogenesis, which are central processes in accelerating progression to advanced fibrosis, cirrhosis, and hepatocellular carcinoma.^3^ In addition to the mechanisms mentioned earlier, alcohol use has been shown to have numerous adverse health consequences, including injury to the brain, heart, pancreas, and liver.^4^

Additionally, patients with MetALD and MASLD were found to have higher cardiovascular risk than those without those diagnoses.^5^ It has also been shown that patients with any cardiometabolic risk factor (CMRF) and increased alcohol consumption (MetALD) had an increased risk of all-cause mortality, cancer mortality, and liver mortality.^6^ Patients with MetALD and ALD had a higher incidence of adverse liver outcomes and all-cause mortality than those with MASLD, along with a similar risk of MACE.^7^ Despite the growing recognition of MetALD as a high-risk entity, the fibrosis burden in those who consume alcohol below the MetALD threshold and carry concurrent metabolic risk factors remains limited.

Interestingly, the prevalence of significant liver fibrosis in the individuals with metabolic dysfunction who consume alcohol below these levels, a “pre-MetALD” population, remains unknown. In this study, we estimated the burden of significant liver fibrosis within this pre-MetALD population (adults consuming 7–14 drinks per week) using a nationally representative sample from the National Health and Nutrition Examination Survey (NHANES) using the 2021–2023 data.

## Methods

### Study Design

This was a cross-sectional analysis of publicly available data from the NHANES from the 2021–2023 cycle.^8^ The NHANES uses a sampling design to produce nationally representative estimates of the U.S. population. The survey involves standardized interviews, physical examination, and laboratory assessments.^8^ The data are de-identified and publicly available; as such, this study did not require institutional review board approval.

### Study Population

Adults aged ≥18 years from the 2021–2023 NHANES cycle were included in the study who met the following inclusion criteria: alcohol consumption of 7–14 drinks per week (below the MetALD threshold), overweight or obesity defined by a body mass index (BMI) ≥25 kg/m^2^ or ≥23 for Non-Hispanic Asians, per the 2023 Delphi consensus criteria for MASLD, valid liver stiffness measurement (LSM) with an interquartile range (IQR)-to-median ratio (IQR/M) ≤0.30,^9^ and complete data for Fibrosis-4 index (FIB-4) score calculation. Participants with any missing data were excluded from the study. Weekly alcohol consumption was calculated from NHANES dietary questionnaire data by multiplying self-reported drinking frequency by average drinks per occasion. Participants were categorized as low-moderate alcohol users (7–10 drinks per week) or high-moderate (11–14 drinks per week).

Using the National Institute on Alcohol Abuse and Alcoholism standard of 14 g of pure alcohol per US drink, the low-moderate tier (7–10 drinks per week) corresponds to 98–140 g per week and the high-moderate tier (11–14 drinks per week) corresponds to 154–196 g per week. These are both below the MetALD threshold for men (≥210 g per week). A subset of women in the high-moderate tier (n = 28) had exposures of 154–196 g per week which approached the female threshold of ≥140 g per week, which is acknowledged as a limitation.

### Cardiometabolic Risk Factor Scoring

CMRF burden was scored per the 2023 Delphi consensus for MASLD.^1^ The first CMRF assessed was overweight or obesity, which was required for cohort inclusion. We then assessed if participants had dysglycemia and hypertension. For dysglycemia, we used HbA1c ≥5.7% (prediabetes or diabetes), self-reported physician diagnosis of diabetes, current insulin use, and current oral diabetic medication use. Hypertension was defined as a mean systolic blood pressure ≥130 mmHg or mean diastolic blood pressure ≥80 mmHg, self-reported physician diagnosis of hypertension, or current antihypertensive medication use. CMRF burden was scored as follows: 1 CMRF (overweight/obesity alone), 2 CMRFs (overweight/obesity plus dysglycemia or hypertension), or 3 CMRFs (overweight/obesity, dysglycemia, and hypertension). Dyslipidemia and waist circumference, which are also recognized components of the 2023 Delphi MASLD criteria, were not incorporated into the CMRF burden score.

### Outcomes

Liver stiffness was measured using vibration-controlled transient elastography (VCTE). The primary outcome was significant liver fibrosis (LSM ≥8 kPa) per American Association For the Study of Liver Diseases (AASLD) two-tier screening algorithm.^10^ Our secondary outcome was advanced liver fibrosis (LSM ≥12 kPa). The FIB-4 was calculated where scores ≥1.3 were defined as elevated and that ≥2.67 was defined as high.^11^ We measured the discordance between FIB-4 and elastography with participants who had an LSM ≥8 but a normal FIB-4 score (≤1.3).

In a pre-specified sensitivity analysis, we applied age-adjusted FIB-4 thresholds (≥2.0 for participants aged ≥65 years and ≥1.3 for participants aged less than 65 years) to assess whether age-related FIB-4 elevation accounted for the observed discordance.

### Statistical Analysis

We accounted for the complex, multistage sampling design of NHANES; therefore, survey examination weights were applied to produce nationally representative estimates. When a stratum contained only one sampling unit after cohort restriction, variance was estimated using a standard adjustment method recommended by the National Center for Health Statistics to ensure stable estimates in small analytic samples.^12^

Survey-weighted prevalence of the primary and secondary outcomes was calculated overall and by CMRF group and alcohol tier. We used multivariable logistic regression to examine associations between CMRF burden, alcohol tier, and significant fibrosis adjusted for age and sex. We excluded race/ethnicity from multivariable models due to insufficient subgroup sizes for stable estimation. All analyses were performed using R software (R Foundation for Statistical Computing, Vienna, Austria).

## Results

Out of the 11,933 participants, 193 met inclusion criteria. From that cohort, we identified 84 participants who had a BMI ≥30. The mean age of the cohort was 55.2 years (standard deviation [SD]: 15.4), and 32.6% were female; 72.3% of the respondents were Non-Hispanic White, 12.0% Non-Hispanic Black, 8.9% Other Hispanic, 4.2% Non-Hispanic Asian, and 2.6% Mexican American. The mean BMI of the cohort was 30.6 kg/m^2^ (SD: 5.1) of which 43.5% were obese, 54.9% were overweight, and 1.6% overweight Asian. The mean alcohol consumption was 9.7 drinks per week (SD: 2.3) where 47.2% were low to moderate alcohol users and 52.8% were high-moderate alcohol users; 30.1% of the participants had dysglycemia, whereas 56.5% had hypertension. With regard to CMRF distribution, 31.1% had 1 CMRF only, 51.3% had 2 CMRFs, and 17.6% had 3 CMRFs. The mean LSM was 6.84 kPa (SD: 6.95), FIB-4 score was ≥1.3 in 38.9% and ≥2.67 in 4.7% of participants (Table 1).

**Table 1:** Baseline Characteristics of Moderate Drinkers With Cardiometabolic Risk Factors, NHANES 2021–2023 (N = 193).

Characteristic	Overall (N = 193)
Age, mean (SD), years	55.2 (15.4)
Female, %	32.6
Male, %	67.4
**Race/ethnicity, %**	
Non-Hispanic White	72.3
Non-Hispanic Black	12
Other Hispanic	8.9
Non-Hispanic Asian	4.2
Mexican American	2.6
**BMI**	
Mean (SD), kg/m^2^	30.6 (5.1)
Overweight (25–29.9), %	54.9
Overweight Asian (23–24.9), %	1.6
Obese (≥30), %	43.5
**Alcohol consumption**	
Mean (SD), drinks/week	9.7 (2.3)
Low-moderate (7–10 drinks/week), %	47.2
High-moderate (11–14 drinks/week), %	52.8
**Cardiometabolic risk factors**	
Dysglycemia, %	30.1
Hypertension, %	56.5
1 CMRF (overweight/obesity only), %	31.1
2 CMRFs, %	51.3
3 CMRFs, %	17.6
**Fibrosis markers**	
LSM, mean (SD), kPa	6.84 (6.95)
FIB-4 ≥1.3, %	38.9
FIB-4 ≥2.67, %	4.7

Data are presented as mean (SD) or percentage.

BMI, body mass index; CMRF, cardiometabolic risk factor; FIB-4, Fibrosis-4 index; LSM, liver stiffness measurement; NHANES, National Health and Nutrition Examination Survey; SD, standard deviation.

Survey weights were applied to produce nationally representative estimates.

The survey-weighted prevalence of significant fibrosis was 15.8% (95% confidence interval [CI]: 9.2–22.4%). Fibrosis prevalence was similar across CMRF burden (1 CMRF: 15.0%, 2 CMRFs: 16.4%, 3 CMRFs: 15.4%), suggesting alcohol exposure rather than metabolic factors may be the primary driver at this consumption level. A dose-response trend was observed where 13.2% (95% CI: 5.5–21%) of low-moderate alcohol users had fibrosis prevalence compared to 18.0% (95% CI: 9.9–26%) among high-moderate alcohol users (Table 2).

**Table 2:** Prevalence of Significant and Advanced Liver Fibrosis by Cardiometabolic Risk Factor Burden and Alcohol Intake.

Subgroup	N	Significant fibrosis (LSM ≥8 kPa)	95% CI	Advanced fibrosis (LSM ≥12 kPa)	95% CI
**Overall**	193	15.80%	9.2 to 22.4%	4.50%	1.9 to 7.1%
**By CMRF burden**					
1 CMRF	60	15.00%	3.0 to 27.0%	2.80%^∗^	−1.3 to 6.8%
2 CMRFs	99	16.40%	7.8 to 25.0%	4.40%^∗^	0.4 to 8.4%
3 CMRFs	34	15.40%	2.4 to 28.5%	8.20%^∗^	0.2 to 16.3%
**By alcohol intake**					
7-10 drinks per week	91	13.20%	5.5 to 21%	3.2%^∗^	−1.0 to 7.4%
11-14 drinks per week	102	18.00%	9.9 to 26%	5.7%	2.2 to 9.2%
**Sensitivity analysis**					
Obese only (BMI ≥30)	84	23.20%	11.9 to 34.4%	5.60%	0.8 to 10.5%

Significant fibrosis was defined as an LSM ≥8 kPa; advanced fibrosis defined as an LSM ≥12 kPa. CMRF burden was scored as follows: 1 CMRF (overweight/obesity), 2 CMRFs (overweight/obesity +dysglycemia or hypertension), and 3 CMRFs (all three). All estimates are survey-weighted.

BMI, body mass index; CI, confidence interval; CMRF, cardiometabolic risk factor; LSM, liver stiffness measurement.

Estimates marked with an asterisk are based on small unweighted event counts and should be interpreted cautiously.

With regard to advanced fibrosis, the prevalence was 4.5% (95% CI: 1.9–7.1) overall. A directional increase was observed in advanced fibrosis prevalence with increasing CMRF burden: 2.8% (95% CI: −1.3 to 6.8%; n = 2) with 1 CMRF, 4.4% (95% CI: 0.4–8.4%; n = 6) with 2 CMRFs, and 8.2% (95% CI: 0.2–16.3%; n = 4) with 3 CMRFs. There was also a similar dose-response trend by alcohol consumption tier: 3.2% (95% CI: −1.0 to 7.4%) among low-moderate drinkers and 5.7% (95% CI: 2.2–9.2%) among high-moderate drinkers. This was consistent with the dose-response pattern observed for significant fibrosis. Given the small number of events, the estimates should be interpreted as directional only (Table 2).

Among the 75 participants who had elevated FIB-4 scores, 26% had an LSM ≥8 kPa. On the other hand, among 31 participants with an LSM ≥8 kPa, 40.9% had a normal FIB-4, representing cases that would have been missed by serum-based screening alone (Table 3).

**Table 3:** Discordance Between FIB-4 Screening and Elastography-Confirmed Fibrosis.

Screening result	N	LSM ≥8 kPa	Interpretation
FIB-4 ≥1.3 (elevated)	75	26.00%	Confirmed by elastography
FIB-4 <1.3 (normal) among participants with LSM ≥8 kPa	31	40.90%	Missed by FIB-4 screening
FIB-4 adjusted (normal) among participants with LSM ≥8 kPa	31	55.1%	Missed by age-adjusted FIB-4 screening

Normal FIB-4 was defined as <1.3; elevated FIB-4 was defined as ≥1.3. Significant fibrosis was defined as an LSM ≥8 kPa.

FIB-4, Fibrosis-4 index; LSM, liver stiffness measurement.

In a sensitivity analysis with age-adjusted FIB-4 thresholds (≥2.0 for participants aged ≥65 years and ≥1.3 for younger participants), the proportion of elastography-confirmed fibrosis cases missed by FIB-4 screening was 55.1% compared to 40.9% with the standard cutoff. Of the discordant cases, the median age was 43 years and only 36% of discordant cases were 65 and older. These results suggest that age-related FIB-4 elevation does not account for the majority of missed cases (Table 3).

In the obese-only cohort (BMI ≥30, n = 84), weighted prevalence of significant fibrosis was 23.2% (95% CI: 11.9–34.4%) and advanced fibrosis was 5.6% (95% CI: 0.8–10.5%). The results in this cohort were higher than that of the primary cohort and consistent with metabolic dose-response (Table 2).

In multivariable logistic regression adjusted for age and sex, neither CMRF burden (odds ratio [OR]: 1.02, 95% CI: 0.28–3.79) nor alcohol tier (OR: 1.46, 95% CI: 0.62–3.42) reached statistical significance consistent with limited statistical power.

## Discussion

The aim of our study was to analyze the prevalence of significant and advanced liver fibrosis in a population of moderate alcohol users with consumption of 7–14 drinks per week. We additionally stratified the population by CMRF including overweight/obesity, dysglycemia, and hypertension. Overall, our results reveal that 15.8% (about 1 in 6 participants) of the cohort of moderate alcohol users had significant liver fibrosis. Our prevalence is comparable to that observed in the MASLD population which estimates 10–17% in US studies despite our cohort consuming alcohol at levels not currently considered clinically significant.^13^ To our knowledge, this is the first study to characterize fibrosis burden specifically in the “pre-MetALD” population using nationally representative data with elastography confirmation.

When taking into account alcohol dose response, in our cohort of moderate alcohol users with at least 1 CMRF, our results reveal that significant liver fibrosis prevalence increased from 13.2% among alcohol-users drinking 7–10 drinks per week to 18% among those consuming 11–14 drinks per week. Furthermore, advanced fibrosis was seen in 4.5% of the overall cohort. Our results are consistent with prior studies including that by Marti-Aguado *et al.* who demonstrated that moderate alcohol intake in MASLD increases the risk of advanced disease to a level similar to that observed in MetALD.^14^ Our findings are further supported by Aberg *et al.* who also showed that metabolic syndrome and alcohol confer higher risk of severe liver disease in the general population.^15^ Our results concur with Marti-Aguado *et al.* that there may be no safe limit of daily alcohol intake in patients with metabolic syndrome or MASLD or in the “pre-MetALD” population.^14^

More concerningly, 40.9% of individuals with significant fibrosis had a normal FIB-4 score (≤1.3), representing cases that would be missed by serum-based screening alone. This discordance has been highlighted by prior studies where Chang *et al.* found that 10% of individuals classified as low risk by FIB-4 were not classified as low risk by LSM.^16^^,^^17^ Our results exceed those reported by Chang *et al.* The higher miss rate in our cohort is likely due to limitations of FIB-4 screening in populations with obesity and metabolic comorbidities.

To evaluate whether age-related FIB-4 elevation contributed to the observed discordance, we performed a sensitivity analysis using age-adjusted thresholds (≥2.0 for participants aged ≥65 years). The miss rate increased to 55.1% for age-adjusted criteria rather than decreased where the majority of discordant cases (63.6%) were younger than 65 years (median age: 43 years). This indicates that age inflation of FIB-4 screening does not account for the high miss rate. It also supports the interpretation that the performance limitations of FIB-4 screening are likely driven by the effects of obesity and metabolic comorbidities on aminotransferase and platelet levels, independent of age.

To evaluate whether hepatic inflammation rather than fibrosis could explain the elevated LSM in discordant participants, we compared alanine aminotransferase (ALT) and aspartate aminotransferase levels across discordance groups. The median ALT in discordant participants (LSM ≥8 kPa with normal FIB-4) was 26 U/L which was comparable to the concordant group (LSM ≥8 kPa with elevated FIB-4; median ALT: 27 U/L). This result argues against active steatohepatitis as the primary explanation for elevated LSM in discordant cases. We also cannot exclude the possibility that a minority of elevated LSM values reflect early inflammatory injury rather than established fibrosis, a recognized limitation of cross-sectional VCTE without histologic confirmation.

What is unique about our study population is that it falls outside current screening frameworks. Current AASLD guidance for MASLD screening recommends FIB-4 screening followed by elastography.^10^ No such guidelines exist for screening in sub-MetALD drinkers. On the other hand, alcohol-associated liver disease guidelines focus on heavy drinking which is ≥14 drinks per week for women and ≥21 drinks per week for men, whereas our study evaluated liver fibrosis in people who are explicitly below this threshold.^18^ Based on our findings, we support consideration of direct elastography in moderate drinkers with CMRF, bypassing FIB-4 screening given its poor performance in this group.

Additionally, our results highlighted that fibrosis prevalence was similar across CMRF groups (15%→16.4%→15.4%) while the alcohol dose-response remained evident. This pattern suggests that alcohol may be the dominant driver for fibrosis rather than incremental metabolic burden. Our findings are consistent with those of Aberg *et al.* where alcohol and metabolic factors have independent and modifying effects where the contribution of the factor varies by exposure level.^15^ However, the interpretation that alcohol is the sole driver should be tempered by several observations. For advanced fibrosis (LSM ≥12 kPa), we did observe an incremental response (2.8%→4.4%→8.2%) and fibrosis prevalence in the obesity-only sensitivity cohort (23.2%) substantially exceeded the primary cohort estimate (15.8%). This suggests that metabolic burden may modulate hepatic risk, particularly at higher fibrosis stages. This pattern is consistent with findings of Aberg *et al.* and Marti-Aguado *et al.* who demonstrated synergistic effects of alcohol and metabolic dysfunction in driving liver disease progression. These findings do not support a single-factor explanation but instead suggest that alcohol and metabolic dysfunction act synergistically in the pre-MetALD population.^14^^,^^15^

### Limitations

There are several limitations with our study. Given the cross-sectional design, it prevents establishing causality of fibrosis progression over time. There is also concern for under-reporting bias, given the respondents self-report alcohol intake.

Additionally, our CMRF burden score was limited to overweight/obesity, dysglycemia, and hypertension, consistent with the primary criteria used in prior NHANES-based MASLD literature. While dyslipidemia and waist circumference are also recognized components of the 2023 Delphi MASLD criteria, we did not incorporate this into the burden score as inclusion of these factors may have reclassified some participants and altered subgroup prevalence estimates. Future analyses incorporating lipid-based CMRF criteria may refine risk stratification in this population.

Moreover, a subset of females in the high-moderate alcohol tier (n = 28, 154–196 g per week) had estimated weekly alcohol exposures approaching the female MetALD threshold of 140 g per week. While all participants were classified based on the drink count below the current MetALD cutoff, some females could represent a borderline exposure population. This did not alter the primary findings as the overall cohort remained below the Delphi MetALD threshold. A sensitivity analysis that excluded these participants also did not change the direction or magnitude of results.

Lastly, our sample size was modest, which limited power for subgroup analyses, and the directional CMRF-advanced fibrosis relationship did not reach statistical significance. Additionally, VCTE-based LSM may be elevated by active hepatic inflammation or congestion, and histologic confirmation was not available in this population-based dataset.

Overall, our study reveals that liver fibrosis affects approximately 1 in 6 adults with CMRF who consume alcohol below the MetALD threshold. The dose-response relationship between alcohol intake and fibrosis prevalence (13.2% in low-moderate vs. 18.0% in high-moderate users) suggests that alcohol consumption at these levels confers meaningful hepatic risk when combined with metabolic dysfunction. However, the incremental trend in advanced fibrosis across CMRF groups and the higher prevalence in the obesity subgroup indicate that metabolic burden modulates risk independently, particularly at higher fibrosis stages. These findings do not support a single-factor explanation and instead suggest that alcohol and metabolic dysfunction act synergistically in the pre-MetALD population.

Additionally, 40.9% of individuals with significant fibrosis had normal FIB-4 scores. This miss rate was not attributable to age-related FIB-4 inflation (age-adjusted sensitivity analysis yielded a higher miss rate of 55.1%) or to hepatic inflammation (median ALT was comparable between discordant and concordant participants). These findings support consideration of elastography as a first-line screening tool in moderate drinkers with CMRF, bypassing FIB-4 screening given its poor performance in this group. By recognizing this “pre-MetALD” population, earlier intervention may be facilitated to prevent progression to MetALD and advanced liver disease.

## Credit authorship contribution statement

Concept and design: Urmimala Chaudhuri. Data acquisition: Urmimala Chaudhuri. Data analysis and interpretation: Urmimala Chaudhuri. Drafting of the manuscript: Urmimala Chaudhuri, Makul Sharma, Erin Currey, and Sangeeta Agrawal. Critical revision of the manuscript for important intellectual content: Urmimala Chaudhuri, Makul Sharma, Erin Currey, and Sangeeta Agrawal. Final approval of the manuscript: all authors.

## Data availability

The data underlying this study are publicly available from the National Health and Nutrition Examination Survey.

## Funding

None declared.

## Declaration of competing interest

The authors declare that they have no known competing financial interests or personal relationships that could have appeared to influence the work reported in this paper.
